# Mood Disturbances Across the Continuum of Care Based on Self-Report and Clinician Rated Measures in the interRAI Suite of Assessment Instruments

**DOI:** 10.3389/fpsyt.2022.787463

**Published:** 2022-05-02

**Authors:** John P. Hirdes, John N. Morris, Christopher M. Perlman, Margaret Saari, Gustavo S. Betini, Manuel A. Franco-Martin, Hein van Hout, Shannon L. Stewart, Jason Ferris

**Affiliations:** ^1^School of Public Health Sciences, University of Waterloo, Waterloo, ON, Canada; ^2^Hebrew Senior Life, Boston, MA, United States; ^3^SE Research Centre, SE Health and Lawrence S Bloomberg Faculty of Nursing, University of Toronto, Toronto, ON, Canada; ^4^Salamanca University and Zamora Hospital, Salamanca, Spain; ^5^Department of General Practice and Medicine for Older Persons, Amsterdam Public Health Research Institute, Amsterdam University Medical Center, Vrije Universiteit Amsterdam, Amsterdam, Netherlands; ^6^Faculty of Education, Western University (Canada), London, ON, Canada; ^7^Centre for Health Services Research, Faculty of Medicine, The University of Queensland, Brisbane, QLD, Australia

**Keywords:** mood disturbance, validity, scale development, reliability, continuum of care, interRAI

## Abstract

**Background:**

Mood disturbance is a pervasive problem affecting persons of all ages in the general population and the subset of those receiving services from different health care providers. interRAI assessment instruments comprise an integrated health information system providing a common approach to comprehensive assessment of the strengths, preferences and needs of persons with complex needs across the continuum of care.

**Objective:**

Our objective was to create new mood scales for use with the full suite of interRAI assessments including a composite version with both clinician-rated and self-reported items as well as a self-report only version.

**Methods:**

We completed a cross-sectional analysis of 511,641 interRAI assessments of Canadian adults aged 18+ in community mental health, home care, community support services, nursing homes, palliative care, acute hospital, and general population surveys to develop, test, and refine new measures of mood disturbance that combined clinician and self-rated items. We examined validity and internal consistency across diverse care settings and populations.

**Results:**

The composite scale combining both clinician and self-report ratings and the self-report only variant showed different distributions across populations and settings with most severe signs of disturbed mood in community mental health settings and lowest severity in the general population prior to the COVID-19 pandemic. The self-report and composite measures were strongly correlated with each other but differed most in populations with high rates of missing values for self-report due to cognitive impairment (e.g., nursing homes). Evidence of reliability was strong across care settings, as was convergent validity with respect to depression/mood disorder diagnoses, sleep disturbance, and self-harm indicators. In a general population survey, the correlation of the self-reported mood scale with Kessler-10 was 0.73.

**Conclusions:**

The new interRAI mood scales provide reliable and valid mental health measures that can be applied across diverse populations and care settings. Incorporating a person-centered approach to assessment, the composite scale considers the person's perspective and clinician views to provide a sensitive and robust measure that considers mood disturbances related to dysphoria, anxiety, and anhedonia.

## Introduction

Mood disturbances, including symptoms associated with anxiety and depression, have been increasing in prevalence in the population (1–3), and this has been exacerbated greatly due to lockdowns, social isolation, economic and health-related concerns during the COVID-19 pandemic (4–6). Problems of mood may be transitory, but they affect persons of all ages, gender, social class, and cultures. They are experienced worldwide, can begin in early stages of life (7–14), and can persist throughout the life course (15–18). Although there are important biomedical factors that can affect mood, a broad array of psychosocial and environmental factors can also trigger, worsen, or prolong these symptoms to become more persistent problems related to depression (15, 19).

As a consequence, mood disturbance is a pervasive problem affecting the general population as well as the subset of persons utilizing health care services. Indeed, health service providers in community and facility-based settings encounter persons with mood disturbance that can complicate the provision of health services (16, 20–23). Mood disturbance is a health problem that must be addressed for its own sake; however, it also warrants attention because it can also interfere with adherence to treatment regimens (24–26), recognition and response to symptoms (27, 28), and it increases resource intensity after adjusting for other clinical factors (29–31). Problems with mood may often be transient; however, they should be attended to with early interventions when the risk of transition to a more permanent form of mood disorder is evident.

The relative success of health care organizations in managing and alleviating problems related to mood is considered to be sufficiently important to justify its use as an outcome based indicator of quality of care in mental health (32) and non-mental health settings (33–39). For example, the Canadian Institute for Health Information publicly reports a risk-adjusted quality indicator for worsened depressive mood in long-term care facilities on a national basis (www.yourhealthsystem.cihi.ca).

The interRAI suite of clinical assessment instruments is used internationally as a comprehensive, integrative health information system providing a common clinical language for evaluating the strengths, preferences, and needs of persons of all ages across the continuum of care (40–48). These instruments include a variety of measures of mood that can be used as standalone items or in summary scales. The most widely used mood measure from this suite is the Depression Rating Scale (DRS) (16, 49–53), which is an additive scale based on the frequency of occurrence of seven items (e.g., tearfulness, repetitive anxious complaints) with scores ranging from 0 to 14. Cross-sector studies of the reliability of the DRS have shown it to have acceptable internal consistency based on Cronbach's alpha scores of 0.70 or more (54–56) as well as strong inter-rater reliability based on weighted kappa values in excess of 0.60 (41, 57).

Although the DRS has been in widespread use for over two decades, there are some important limitations that warrant efforts to develop an alternative mood measure that could be employed across health settings over the life course. For example, the tripartite model of depression and anxiety (58, 59) suggests that it is important to consider indicators of dysphoria, anxiety, and anhedonia. However, the DRS includes only indicators of the first two factors, despite the relevance of anhedonia (60), and longitudinal studies have shown that items on social withdrawal provide additional predictive value for future depression diagnoses after adjusting for the DRS (61). A further criticism has been related to the modest correlation between the clinician-rated DRS with self-reported measures like the Geriatric Depression Scale (GDS) (62). Koehler et al. showed that the DRS and GDS were both related to depression diagnoses in long-term care settings (62) yet, they were relatively uncorrelated with each other. This limited correlation suggests that clinician-rated and self-reported measures address important, but different aspects of mood. Clinicians may be insensitive to certain aspects of mood that self-report measures may pick up. In addition, practical and economic considerations may preclude the use of clinician-only rated systems for screening of the general population. For example, some low resource nations do not have sufficient mental health human resources to respond to the clinical needs of all persons with severe mental health issues (63, 64) let alone to have health professionals to do broad-based population screening. There is also growing interest in the use of self-report measures for patient reported outcome measurement (65, 66). On the other hand, self-report measures may also cause under-detection due to cultural biases (67, 68) or non-response due to cognitive impairment or communication difficulties (62). Hence, there could be important advantages to an assessment strategy that combines the use of clinician-rated and self-reported measures of mood that are consistent between populations, health and social service settings, and geographic regions, in a manner that permits longitudinal monitoring of mental health outcomes. Such measures could be used for person-level and organizational-level applications including care planning, need identification, outcome evaluation, measurement-based care, and risk adjustment.

The DRS was first created as a clinician-rated scale with interRAI's original nursing home assessment (50) and then validated for use with assessments for other settings as they became available (51–54). However, with the advent of interRAI's new integrated suite of instruments, a set of three self-reported measures of dysphoria, anxiety, and anhedonia was introduced. Our objective was to create alternative mood scales for use with all interRAI assessments including a composite version with both clinician-rated and self-reported items as well as a self-report only version. Testing was also done to ensure that the clinician-rated items could function as a scale in legacy instruments that predate the new suite (e.g., RAI-MDS 2.0; RAI-MH); however, our focus here is on the newer instruments since they will be the only standard to be adopted internationally in any new implementations of interRAI systems.

## Methods

We completed a cross-sectional analysis of 511,641 interRAI assessments of Canadian adults age 18 years or more in community mental health, home care, community support services, nursing homes, palliative care, acute hospital, and general population surveys to develop, test, and refine new measures of mood that combined clinician and self-rated items. We examined convergent validity, criterion validity, and internal consistency across a continuum of care settings serving diverse populations. Although interRAI data are available for over 30 other countries, we chose to focus on Canadian data only to avoid country level effects and as such we defer analyses of those international data for future testing. Our emphasis here was on multiple care sectors within one nation with awareness that cross-national testing will be an important next step.

### Samples

This study includes stratified analyses of large samples of individuals with highly diverse demographic backgrounds and heterogeneity in health status across multiple care settings, age groups and life stages. The data for our study samples came from three types of implementations of interRAI systems First, we sampled from diverse settings where there was mandated routine clinical use in the full population of service recipients: home care^1^ (using interRAI Home Care (69), community support services [using interRAI Community Health Assessment (56)], palliative home care [using interRAI Palliative Care (70–72)], and nursing homes [using interRAI Long Term Care Facility (73, 74)]. Most of these data are from the province of Ontario; however, that province still uses an older version of the nursing home instrument^2^ (75, 76) that excludes self-report items so data from the province of New Brunswick based on the newer interRAI LTCF assessment were used instead. Second, pilot or regional implementations of interRAI systems were done for community mental health services [using interRAI Community Mental Health (44)], wellness checks in home care [using the interRAI Check-Up Self-report version (77, 78)], and emergency department screening [using the interRAI Emergency Department Contact Assessment (79)] of older adults in acute hospitals. The third type of implementation was research-only use of interRAI self-report items in telephone and on-line surveys of the general population.

Table 1 provides an overview of the seven main study populations used in our analyses. In every setting we used only the most recent observation for each person assessed, so the within-sector samples all represent unique individuals. There is a possibility that some persons were assessed at different times in different settings (e.g., home care and nursing homes), but we did not have identifiers that could be used to link records between sectors. Therefore, although there were up to half a million individuals included in this analysis, the actual number will be somewhat less than that because of some persons receiving care in two or more settings during the study period.

**Table 1:** Sample characteristics.

**Characteristic**	**CMH** ** (** ***n*** ** = 7,256)**	**HC** ** (** ***n*** ** = 352,161)**	**CHA** ** (** ***n*** ** = 28,302)**	**CUSR** ** (** ***n*** ** = 4,930)**	**LTCF** ** (** ***n*** ** = 8,237)**	**PC** ** (** ***n*** ** = 106,759)**	**EDCA** ** (** ***n*** ** = 1,432)**	**Community surveys**	
								**Telephone (*n* = 643)**	**On-line (*n* = 1,921)**
Region	Ontario	Ontario	Ontario	Ontario	New Brunswick	Ontario	Ontario, Quebec	Waterloo Region	Canada
Setting	Community mental health	Long-stay home care	Community supports	Home care wellness check	Nursing homes	Palliative home care	Emergency departments	General population	General population
Basis for use	Regional implementation	Provincial mandate	Provincial mandate	Regional implementation	Provincial mandate	Provincial mandate	Research pilot	Research	Research
Years	2005–2019	2018–2021	2016–2017	2020–2021	2016–2020	2011–2021	2017–2018	2011	2021
**Age**									
18–44	55.0	2.8	3.1	3.7	0.5	2.6	0.0	37.2	49.5
45–64	34.0	11.5	10.4	15.0	4.8	24.1	0.0	38.8	34.7
65–74	6.2	16.2	16.6	19.4	12.4	26.3	12.4	|	12.0
75–84	3.3	29.5	31.7	27.8	28.4	27.7	38.6	|24.0	2.4
85+	1.5	40.0	38.1	34.0	54.0	19.2	49.1	|	0.4
Female	52.1	60.5	68.4	59.9	65.4	41.6	58.9	59.6	56.7
Married	29.3	37.7	26.7	37.9	28.8	60.0	NA	NA	57.6
Depression/ mood diagnosis	54.2	24.0	18.6	NA	27.5	NA	NA	NA	34.2
CPS							NA	NA	NA
0	67.5	19.0	44.1	33.7	6.7	53.2			
1–2	28.8	55.1	47.1	50.1	31.4	36.2			
3–6	3.7	25.8	8.7	16.2	61.9	10.7			

*The 2011 Waterloo Region general population survey did not have adequate sample size to allow breakdown of older adults into further subgroups. The reported value reflects the percentage aged 65+ years in that survey only*.

The community mental health sample was of 7,256 adults receiving those services in the Niagara and Chatham Kent regions of Ontario between 2015 and 2019. Most of this sample was comprised of young and middle-aged adults with only 11.0% being aged 65 years or more. About half were male and about one-third were married^3^. As people within this sample were accessing community-based mental health services, it is not surprising that more than half had a mood disorder diagnosis present when assessed. Less than 5 percent had moderate or worse cognitive impairment based on a score of three or more on the Cognitive Performance Scale (80, 81), which is substantially lower than would be evident in care settings for frail older adults (e.g., home care, nursing homes).

Adult home care clients comprised the largest study sub-population with 352,161 unique Ontarians receiving long-stay home care services between 2018 and 2021. These services predominantly target older adults, so most of the sample was over 65 (85.7%), most were female, and one-third were married. About one quarter had an existing depression diagnosis, and about one quarter had moderate or worse cognitive impairment.

The community support service sub-population included 28,302 Ontarians receiving community services, representing a lighter care population than is typically seen in home care. The most recent data available were for the 2016–2017 period. This population has a similar age distribution as seen in the long-stay home care population, but somewhat more females, fewer married individuals, lower rates of depression diagnoses, and notably lower rates of moderate or worse cognitive impairment.

A second long-stay home care population sample was comprised of 4,930 clients captured during 2020–2021. This sample, who normally would have received the interRAI HC assessment as part of routine care, were screened with the interRAI Check Up self-report instrument (and not the interRAI HC instrument) due to practical restrictions that prevented in-person visits during the start of the COVID-19 pandemic. As shown in Table 1, this sub-population was comparable to the interRAI HC assessed population in age, marital status, and gender but they had lower rates of cognitive impairment.

The nursing home sample was the only sample to be fully gathered outside of Ontario, since New Brunswick was the only Canadian province to have fully adopted the interRAI LTCF at the start of our study. The sub-population of 8,237 unique individuals were assessed between 2016 and 2020 and they had the highest proportion of persons aged 85 years or more. Two thirds were female and about one quarter were married. Diagnosed depression was evident at a rate comparable to the Ontario home care sample; however, this population also had the highest rates of moderate or worse cognitive impairment.

The palliative care sample was comprised of 106,759 unique Ontarians receiving community based palliative care through the provincial home care program between 2011 and 2021. This sub-population was younger than the home care population; however, about three quarters were aged 65 years or more. Unlike the home care population, the majority were married and only about 40 percent were female. Depression diagnoses were not available, but moderate or worse cognitive impairment affected only about 11 percent of this sub-population.

The emergency department sample of 1,432 individuals was obtained from a pilot implementation of the interRAI ED-CA in a study of screening for potential frailty among older adults in emergency departments in Ontario and Quebec done between 2017 and 2018. The study sample was constrained to older adults with about half being aged 85 years or more. Marital status, depression diagnoses, and CPS scores are not tracked in the ED-CA.

Two community samples of the general population were obtained from research projects done before and during the COVID-19 pandemic. One study included 642 respondents to a telephone survey in the Waterloo Region only that was done in 2011. Participants were selected using random-digit dialing of the general population. A second study done in partnership with Mental Health Research Canada used an on-line survey of a polling company's pre-existing participant pool, and it included 1,921 respondents from across Canada in February 2021, which corresponded to the third wave of COVID-19 in Canada. Both samples are comprised mainly of young and middle-aged adults with comparable percentages who were female. As with the palliative sample, the majority of those in the on-line survey were married (not available for the 2011 survey). Of particular note is the high rate of depression diagnoses reported in the 2021 community survey with rates exceeded only by the community mental health sample (question was not asked in the 2011 survey).

### Measures

Five of the interRAI assessments used in this research (CMH, HC, CHA, LTCF, PC) are comprehensive assessments completed by trained health professionals (mainly nurses) at different points during the episode of care (40, 43). Typically, this occurs at admission/intake and then on a structured reassessment cycle that varies by sector (e.g., 3 months in long-term care and 6 months in home care or community mental health). In addition, periodic reassessments may occur on an unscheduled basis if there is a recognized clinically significant change (improvement or worsening) that is persistent and requires a change to the care plan. All of these instruments have multiple applications for diverse audiences including care planning, outcome measurement, quality monitoring, and resource allocation (32, 43, 44, 82).

The interRAI ED-CA is a clinician-led screening-level assessment that is done with older adults in emergency departments. It is not intended to support the full care planning process; however, it includes several measures that can be used to inform clinical management in the emergency department (79, 83, 84).

For the clinician-led assessment and screening instruments, the assessor employs evidence from all sources of information to determine the most appropriate response for a given item based on their best judgement. This includes direct observation of and interviews with the person, discussion with key informants (e.g., family members when appropriate), information provided by staff and professional communications, and review of the chart. Clinicians are provided with standardized item definitions, inclusion/exclusion criteria, illustrative examples, observational timeframes, and coding guidelines as part of the standard training approach for interRAI assessments (85, 86). Most items on the clinician-led assessments are based on clinical judgement; however, there is a subset of items that are self-report only with standardized narrative structures for the items and response sets. The cross-sector reliability and validity of these instruments have been reported elsewhere (41, 54, 57). The composite measure of mood that we developed is based on a combination of clinician-rated and self-reported items included in these assessment systems; however, we also developed a self-report only variant that can be used as a standalone scale. In addition, we created a clinician-only variant that can be used for backward compatibility with legacy instruments (results for clinician-only version not reported here but are available on request).

The Check-Up Self-report version and the survey questions used for the general populations are based on self-report items that have fixed, standardized questions and responses. The CU-SR can be self-administered or be done with a lay survey interviewer, but the interviewer does not require clinical credentials. The responses are strictly based on the person's self-report without clinical judgement being applied by the interviewer. Previous research has reported in the reliability and validity of the CU-SR in community-based research (77, 78).

### Analyses

#### Scale Construction

**Figure 1:**
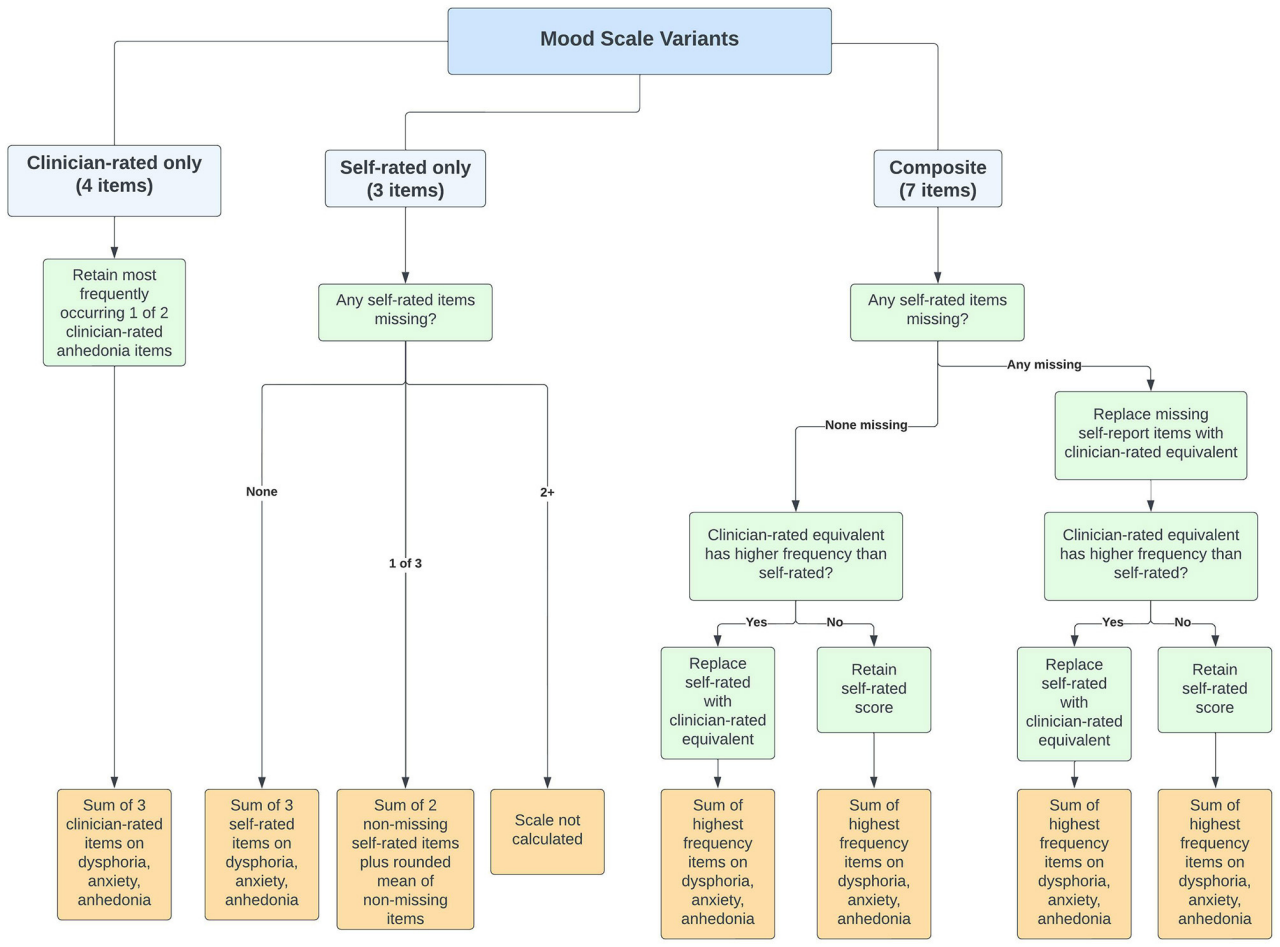
Schematic representation of coding rules for three variants of Mood scale.

Figure 1 provides a schematic representation of coding rules for three variants of mood scales that can be derived from the new interRAI suite of assessments. The Composite Mood Scale (CMS) is based on four clinician-rated and three self-rated items dealing with dysphoria, anxiety, and anhedonia. All indicators use a 3-day lookback period and have the following four level response values: 0-not present; 1-not exhibited, but present recently; 2-exhibited in 1–2 of last 3 days; 3-exhibited daily (wording varies slightly for self-report items). The clinician-rated items include one item for dysphoria (presence of sad, pained, or worried facial expressions), one item for anxiety (repetitive non-health-related anxious complaints), and two items for anhedonia (social withdrawal and loss of interest^4^). The self-report items include one for dysphoria (feeling sad, depressed, or hopeless), one for anxiety (feeling anxious, restless, or uneasy) and one for anhedonia (lost interest in things normally enjoy). The self-rated items allow for a non-response category, but that option is not permitted for the assessor rating. Therefore, missing values should only be an issue with the self-rated items.

We chose to do this to maximize sensitivity of detection of mood disturbance. In creating the composite scale, a variety of coding options were considered to test whether collapsing certain response values (e.g., combining infrequently present and not present values) would improve scale performance and we explored alternative rules for use of self-report vs. clinician ratings. There was no improvement in performance when response value ranges were collapsed. The potential loss of sensitivity was a greater concern, so we left all response values unmodified. For the two clinician rated items on anhedonia, the highest of the two values was used to specify the clinician observation for that indicator. After considering alternative substitution or additive models, we chose to code the composite scale first based on the person's self-report. If the self-report item was missing (e.g., unwilling or unable to respond) it was substituted with the clinician-rated item for the indicator. Next, if the clinician rating indicated greater frequency of a symptom being present, the clinician rating replaced the self-report. Once the value of each composite item was specified for the three indicators, their scores were summed to create a score with values of 0–9. All alternative coding options were examined with respect to their ability to predict outcomes of interest; however, this approach provided the best overall performance (results available on request).

The Self-reported Mood Scale (SMS) simply sums the scores for the three self-report items. A missing value was permitted for one of the three values and compensated for by assigning the rounded value of the mean of the other two items to the third missing item. As with the composite scale, the self-reported scale values ranged from 0 to 9 with higher scores indicating greater mood disturbance.

#### Validity

There were three main approaches used to establish validity of the composite and self-reported mood scales. **First**, we used the CMH data to identify which variant of these scales best predicted presence of two types of indicators of convergent validity: (a) a provisional diagnosis of a mood disorder, indicating clinical designation of the problem meeting diagnostic criteria; (b) self-harm indicators, as non-diagnostic markers of presence of a severe problem. The variant that performed best in those comparisons was examined for its relationship to a depression diagnosis in all other data sets where that diagnosis was available.

Second, we examined evidence of convergent validity by comparing mean scale scores (and 95% confidence limits) against sleep disturbance and depression/mood disorder diagnoses across care settings. The other assessments do not include self-harm indicators; however, a measure of difficulty sleeping is widely available in the interRAI suite and it is known to be associated with mood disturbance (3, 87). The diagnosis items for depression or mood disorder in clinician-rated assessments are based on the clinician's confirmation (using all sources of information available) of the presence of a formal diagnosis made by a physician.

The third approach for the self-reported scale only was to establish convergent validity against the Kessler-10 Psychological Distress Scale (K-10) in the on-line community sample only (K-10 was not available in other data sets). The K-10 measures non-specific psychological stress (rather than a measure of mood disorders) that considers anxiety and depressive symptoms experienced in the last 4 weeks. The K10 is commonly used mental health in population health surveys, including in the World Health Organization World Mental Health Initiative surveys (88), as well as in Australia (89), New Zealand (90), and Canada (91).

#### Reliability

Inter-rater reliability of the clinician-rated items has been reported for the interRAI suite of assessments elsewhere [2, 17] and is not relevant to self-reported items. We did not examine test-retest reliability, but previous multinational work with the interRAI LTCF reported average test-retest reliability of all items with weighted kappas between 0.75 and 0.92 (74). Therefore, our focus was on testing internal consistency based on Cronbach's alpha values for the composite and self-report versions of the scales in all settings.

## Results

Table 2 provides the distributional characteristics, scale reliabilities, correlations between scales, and rates of missing values for the composite and self-report versions of the mood scale across care settings. For both versions of the scale, the highest severity ratings were evident in the community mental health sample. Among the clinical settings, the least severe ratings for both scales were evident in the community support service sample assessed with the interRAI CHA. The only setting where the self-report and composite scales resulted in different rank orders of severity for the sector was in the interRAI LTCF sample. Those able to self-report had scores comparable to the CHA sample; however, when the composite version was used to include ratings for persons with missing data on the self-report items, the mean severity score was second highest after community mental health.

**Table 2:** Mood scale variant means (SD), medians (Q1–Q3), internal consistency, scale correlations, and missing rates by setting.

**Mood scale**	**CMH** ** (*****n*** **= 7,256)**	**HC** ** (** ***n*** ** = 352,161)**	**CHA (** ***n*** ** = 28,302)**	**CUSR (** ***n*** ** = 4,930)**	**LTCF (** ***n*** ** = 8,237)**	**PC (** ***n*** ** = 106,759)**	**EDCA (** ***n*** ** = 1,432)**	**Community surveys**	
								**Telephone (** ***n*** ** = 643)**	**On-LINE (** ***n*** ** = 1,921)**
**Mean (95% CL)**									
Self-Report	3.9 (3.8–3.9)	1.6 (1.6–1.6)	1.3 (1.3–1.3)	2.4 (2.3–2.4)	1.3 (1.3–1.4)	2.1 (2.0–2.1)	2.8 (2.6–3.0)	1.1 (1.0–1.3)	3.5 (3.6–3.4)
Composite	4.8 (4.7–4.9)	2.1 (2.1–2.2)	1.8 (1.8–1.8)	NA	2.5 (2.4–2.6)	2.4 (2.4–2.4)	NA	NA	NA
**Cronbach's alpha**									
Self-Report	0.87	0.64	0.72	0.70	0.75	0.78	0.79	0.65	0.82
Composite	0.90	0.75	0.79	NA	0.78	0.70	NA		NA
Correlation between scale variants	0.92	0.89	0.90	NA	0.77	0.93	NA	NA	NA
**Percentage missing**									
Self-report	3.5%	11.0%	3.6%	0.0%	43.8%	13.5%	25.4%	2.2%	1.2%
Composite	0.6%	0.03%	1.2%	NA	0.6%	0.02%	NA	NA	NA

For the settings where only self-report measures were available (i.e., interRAI Check-Up, telephone and on-line surveys), the severity of mood disturbance was second highest among clinical settings in the emergency department. However, a more striking finding is the substantial difference across the estimates for the general population at two points in time. Mean survey ratings in 2011 compared with during the third wave of the COVID-19 pandemic were very different (1.1 vs. 3.5, respectively).

The Cronbach's alpha values for 12 of the 14 possible scores were above 0.70 with seven instances of scores of 0.78 or more. The only instances where values fell below 0.70 were with self-report in home care (0.64) and the 2011 telephone survey (0.65).

The two variants of the scale are highly correlated with each other where both variants were available. That is to be expected given that self-report items are common to both variants. The main uses of the composite scale are to compensate for missing self-report data and to increase sensitivity by using higher scores from clinicians in cases of disagreement. Therefore, it is not surprisingly, that they are less correlated (*r* = 0.77) in the interRAI LTCF where missing values for the self-report items are most prevalent (about 44% of residents compared with 25% of emergency department patients and 4% of community support clients).

Table 3 shows the unadjusted odds ratios (and 95% CL) for the relationship between scores on the composite and self-reported mood scales against the presence of a physician's diagnosis of mood disorder or depression. For most settings, the odds ratio of such a diagnosis being present increased by about 1.3 for each 1-point increment on the scale. For context, persons with a maximum score of 9 on the scale would have a 12-times greater odds of a physician's mood disorder/depression diagnosis than those in the reference group with a score of 0. The c statistic values ranged between 0.67 and 0.75 with strongest values in the community mental health sample, which is in the range of the conventional 0.7 threshold for a good model (92). The exception was in the interRAI LTCF sample where both scales had weaker performance.

**Table 3:** Unadjusted odd ratios (95% CL) for depression or mood disorder diagnosis present, by mood scale variant and setting.

	**CMH (** ***n*** ** = 7,256)**	**HC (** ***n*** ** = 352,161)**	**CHA (** ***n*** ** = 28,302)**	**CUSR (** ***n*** ** = 4,930)**	**LTCF (** ***n*** ** = 8,237)**	**PC (** ***n*** ** = 106,759)**	**EDCA (** ***n*** ** = 1,432)**	**Community survey on-line (** ***n*** ** = 1,921)**
**Composite scale (1 pt increments)**								
Odds ratio (95% CL)	1.32 (1.30–1.35)	1.26 (1.25–1.26)	1.30 (1.29–1.32)	NA	1.09 (1.07–1.10)	NA	NA	NA
c statistic	0.754	0.671	0.702		0.565			NA
**Self-Report (1 pt increments)**								
Odds ratio	1.33 (1.31–1.36)	1.30 (1.29–1.30)	1.34 (1.33–1.36)	NA	1.12 (1.09–1.15)	NA	NA	1.33 (1.27–1.38)
c statistic	0.744	0.674	0.692		0.57			0.72

Table 4 provides evidence of convergent validity of the two mood scales against mood disorder diagnosis and indicators of self-harm in the community mental health sample. This context is informative because there is greater mental health expertise available and there is greater variance in mental health indicators including higher rates of severe symptoms. We also used these analyses to specify appropriate cut-points for the scales should clinicians wish to use threshold values to inform decision-making. For all four indicators of self-harm and for the mood disorder diagnosis, each increment in the scale had comparable odds ratios (values ranged between 1.23 and 1.35) and c statistics (values ranged between 0.68 and 0.75). Using mood disorder as a marker for an initial cut-point after the baseline 0 value, suggests that appropriate scores would be 4 or more for the composite scale and 3 or more for the self-reported scale. The more severe threshold based on self-harm indicators would be values of 7 for the composite scale and 6 for the self-report version. For example, persons with a score of 7 on the CMS have over 8-times greater odds of having a suicide plan present compared with those with scores of 0.

**Table 4:** Unadjusted odds ratios (95% CL) and optimal scale cut-points based on various mental health markers by mood scale variant in community mental health sample only (*n* = 7,256).

	**Mood disorder**	**Recent self-harm ideation**	**Recent self-harm attempts**	**Others concerned about self-harm**	**Suicide plan present**
**Composite scale (1 pt increments) 0–9 scale**					
Odds ratio (95% CL)	1.32 (1.30–1.35)	1.35 (1.31–1.39)	1.25 (1.20–1.31)	1.27 (1.24–1.31)	1.35 (1.29–1.41)
c statistic	0.754	0.733	0.682	0.707	0.734
**Optmal cut-points**					
Dist to 0,1	4	7	7	7	7
Sens-Spec	4	7	7	7	8
Youden	4	6	4	8	7
**Self-report (1 pt increments) 0–9 scale**					
Odds ratio (95% CL)	1.33 (1.31–1.36)	1.32 (1.29–1.35)	1.23 (1.18–1.28)	1.24 (1.21–1.27)	1.31 (1.26–1.36)
c statistic	0.744	0.739	0.689	0.696	0.734
**Optimal cut-points**					
Dist to 0,1	3	5	5	6	6
Sens-Spec	3	6	6	6	6
Youden	2	5	5	6	6

**Figure 2:**
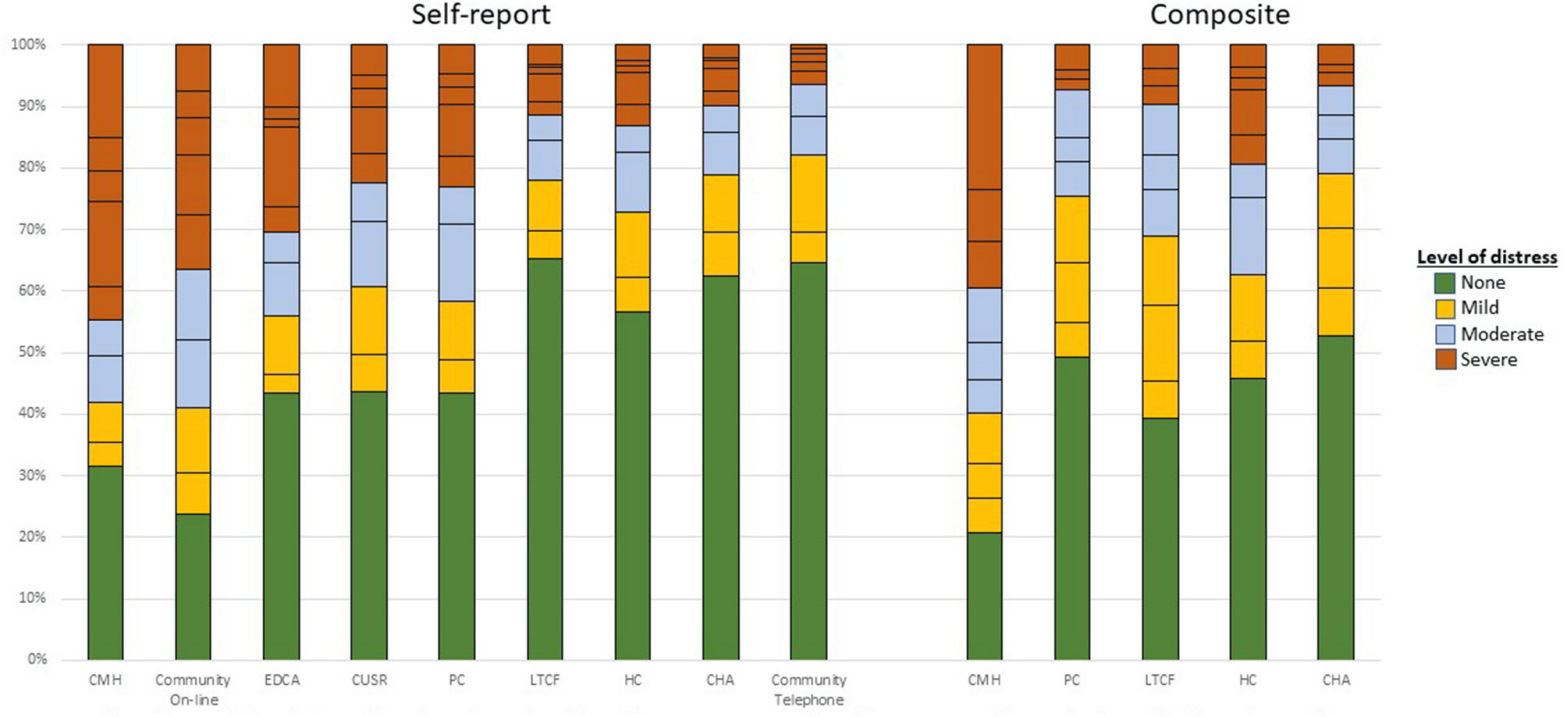
Distribution of mood scale variants by care setting and scale variant. Colors reflects similar levels of mood disturbance for each scale based on cut-points reported in Table 4. Horizontal lines within the stacked bars reflects single point increments in the scale value within each settings. The specific scale values (and text labels) in the stacked bars are: green (none)−0 for both self-report and composite version; orange (mild)−1–2 for self-report and 1–3 for composite; gray (moderate)−3–4 for self-report and 4–6 for composite; and red (severe)−5+ for self-report and 6+ for composite version.

Figure 2 shows the mood scale distributions across settings using the abovementioned cut-off values. There was within-group heterogeneity in these scale scores, but the most severe ratings were found in the community mental health sample. One exception was the on-line general population survey during COVID-19 where the severity scores for the self-report scale were the highest for all settings. Also, as noted earlier, the prevalence of signs of mood disturbance of differing severity varies most greatly in LTCF where those in the self-report and composite scale subsamples differ most substantially. In that setting, the composite mood scale demonstrates substantially higher rates of mood disturbance than does the self-reported variant.

Table 5 shows the relationships of the two scale variants with difficulty sleeping and depression/mood disorder diagnoses across settings based on mean scale scores and 95% CL. For both scales and in all settings, higher frequency of sleep difficulties was associated with significantly higher mean scores in the mood scales. The same was also true for the presence of depression/mood disorder diagnoses. Again, the COVID-19 community sample demonstrated highest scores on these scales for both those with and without depression diagnoses present.

**Table 5:** Evidence of convergent validity of mood scale variants with depression or mood disorder diagnosis present.

	**CMH (** ***n*** ** = 7,256)**	**HC (** ***n*** ** = 352,161)**	**CHA (** ***n*** ** = 28,302)**	**CUSR (** ***n*** ** = 4,930)**	**LTCF (** ***n*** ** = 8,237)**	**PC (** ***n*** ** = 106,759)**	**Community surveys**	
							**Telephone (** ***n*** ** = 643)**	**Telephone (** ***n*** ** = 643)**
**Self-Report**								
**Difficulty sleeping**								
Not present	2.0 (1.9–2.1)	1.2 (1.2–1.2)	0.9 (0.9–0.9)	1.9 (1.8–2.0)	1.2 (1.1–1.2)	1.7 (1.7–1.7)	0.7 (0.5–0.9)	NA
Present, not last 3 days	3.5 (3.3–3.8)	1.8 (1.7–1.8)	1.5 (1.4–1.6)	2.4 (2.0–2.7)	1.4 (1.2–1.6)	2.3 (2.2–2.4)	1.3 (0.8–1.8)	
1–2 of last 3 days	4.9 (4.7–5.1)	2.0 (2.0–2.0)	1.8 (1.8–1.9)	2.8 (2.6–3.0)	2.0 (1.8–2.3)	2.6 (2.5–2.6)	1.2 (0.9–1.6)	
Daily last 3 days	6.4 (6.3–6.5)	2.5 (2.5–2.5)	2.1 (2.1–2.2)	3.7 (3.5–3.8)	2.6 (2.2–3.0)	2.9 (2.9–2.9)	1.9 (1.4–2.3)	
**Depression/Mood disorder diagnosis**								
Not present	1.9 (1.8–2.0)	1.2 (1.2–1.2)	1.0 (1.0–1.0)	NA	1.2 (1.1–1.2)	NA	NA	2.8 (2.6–2.9)
Present	4.8 (4.7–4.9)	2.8 (2.8–2.8)	2.6 (2.6–2.7)		1.8 (1.7–2.0)			5.0 (4.7–5.2)
**Composite**								
**Difficulty sleeping**								
Not present	2.7 (2.6–2.8)	1.7 (1.7–1.7)	1.3 (1.3–1.4)	NA	2.2 (2.1–2.3)	2.0 (2.0–2.0)	NA	NA
Present, not last 3 days	4.7 (4.5–4.9)	2.4 (2.3–2.4)	2.0 (1.9–2.1)		2.8 (2.6–2.9)	2.7 (2.6–2.8)		
1–2 of last 3 days	5.9 (5.7–6.1)	2.7 (2.6–2.7)	2.4 (2.3–2.5)		3.6 (3.4–3.8)	3.0 (3.0–3.1)		
Daily last 3 days	7.5 (7.4–7.6)	3.2 (3.2–3.2)	2.8 (2.7–2.9)		4.2 (3.9–4.4)	3.3 (3.2–3.3)		
**Depression/Mood disorder diagnosis**								
Not present	2.6 (2.5–2.8)	1.7 (1.7–1.8)	1.4 (1.4–1.5)		2.3 (2.3–2.4)	NA	NA	NA
Present	5.8 (5.7–5.9)	3.4 (3.4–3.4)	3.3 (3.2–3.4)		3.0 (2.8–3.1)			

**Figure 3:**
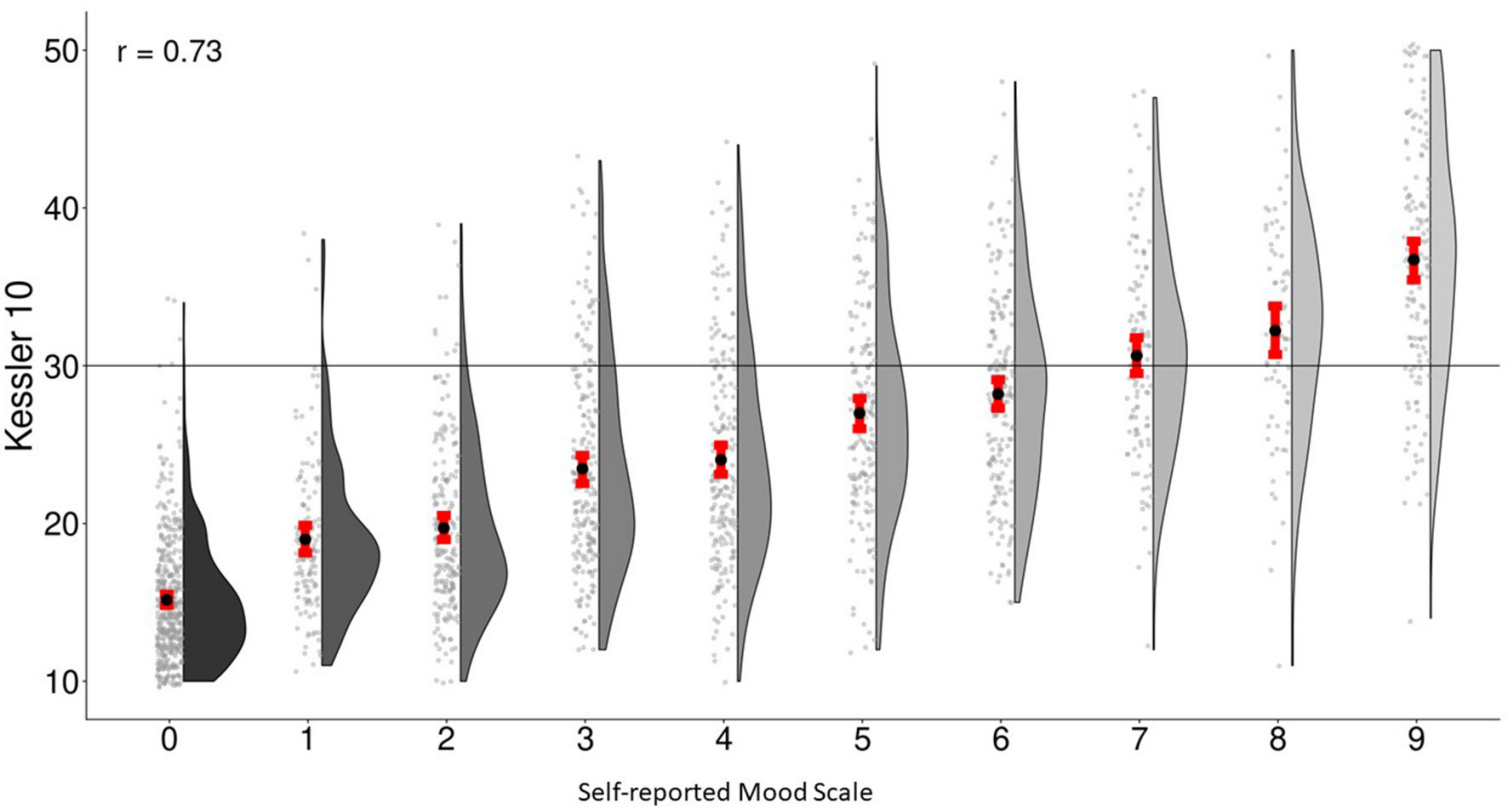
Association between self-reported mood-scale-self-report and Kessler-10 in general-population survey.

Finally, Figure 3 shows the relationship between the K-10 and the SMS. Increments in the SMS are significantly associated with higher K-10 scores and the correlation between the two scales is 0.73. This value is comparable to the value of 0.70 reported elsewhere for brief measures like the PHQ-2 and K-6 (93). Butterworth and colleagues suggest that a K-10 score of 30 or more indicates “very high risk of psychological distress” (1). Persons with the suggested SMS with values of 6 (the cut-off suggest by self-harm indicators in the community mental health sample) and 7 have mean (95% CL) K-10 scores of 28.2 (27.3–29.3) and 30.6 (29.4–31.8), respectively. Put differently, the percentage of persons with K-10 scores increased consistently with each increment of the SMS ranging from 1% of those with a score of 0–85% of those with a score of 9 on the SMS (see Figure 2).

## Discussion

With the transition to the new suite of interRAI assessment instruments (40) the composite and self-reported mood scales can be adopted as an alternative to the previous standard of the Depression Rating Scale (DRS). While the DRS will remain useful for historical trend analyses, these mood scales have several advantages over the DRS. First, with the inclusion of self-report measures they more directly includes the person's perspective than measures that rely on clinician-only assessments. Second, it is designed to be more sensitive than the DRS by using two types different of measures (clinician-rating and self-report) to better capture indications of mood disturbance. This should help in reducing the rate of under-detection of problems with mood. Third, the addition of anhedonia measures fills an important gap in the content validity of the DRS. Although the mood scale deals with a possibly transitory state in mood, having indicators related to anhedonia provides additional evidence relevant to broader aspects of depression if the indicators of mood disturbance persist over time. Fourth, our analyses provide more robust evidence for clinical cut-points in the mood scales than has been available for the DRS. Fifth, the availability of alternative forms of the scale allows for comparability of measures across diverse clinical settings where self-report or clinician-rated only measures may not be possible or desirable. This also gives the opportunity to compare results from the two perspectives separately when the clinician or self-report scales are considered on their own.

Our analyses provide clear evidence of reliability and validity for these scales to be used as a common standard with various adult age groups across settings in the continuum of care. The scales can effectively employ both the person's perspective and clinician ratings in a manner that allow it to be used for comparisons in settings where factors like cognitive impairment may be important barriers to those that rely on self-report alone. The results shown in Table 2 illustrated why having the option of clinician ratings available is essential for settings like home care, nursing homes, and palliative care where item non-response for self-ratings can be substantial. On the other hand, the 3-item self-report scale poses minimal burden while demonstrating solid measurement properties across settings and populations.

The results variations in indicators of mood disturbance across care settings were consistent with what one would expect based on the populations served in those settings. However, it was also interesting to note the stark differences in scale scores in the general population samples prior to and during the COVID-19 pandemic. This shows that the mood scale variants are sensitive to contextual variables and major external events.

One observation of concern was the low c statistics and odds for prediction of depression diagnosis in nursing home settings. This might simply be a function of error variance when computing nine different comparisons; however, it is worth noting that depression is often under-detected in that setting (16, 94). The robust c statistic value in community mental health settings suggest that the problem may be with inadequate recognition in nursing homes rather than with the performance of the scale itself. In addition, lack of access to psychiatrists, facility level characteristics or clinical practices may affect the recognition of symptoms of depression (95).

An important benefit of the mood scale variants is that they can be scaled up for use on a national basis with relative ease where there are existing implementations of interRAI systems. In many countries, large scale adoption of new interRAI assessments is complete or underway. For example, over 20 million interRAI assessments have been completed on over 5 million unique Canadians and there is an existing e-heatlh infrastructure to support front-line clinical use, management, governance, and national policy applications of these systems. Several provinces have already begun transition to the new suite of instruments from older versions, so the inclusion of coding standards for these scales in clinical software will allow for rapid, large-scale deployment. In other countries, like the US, New Zealand, Finland, Belgium, Netherlands, Switzerland, Italy, Hong Kong the adoption of one or more of these newer instruments is complete so conversion to use of this scale becomes a relatively modest undertaking related to information technology and training.

It should be emphasized that the mood scale variants are intended to be a decision support tools that could improve access to necessary mental health services. While they are clearly associated with diagnoses of mood disorders or depression, they are not intended to be a substitute for judgement by mental health professionals. They may be used for screening to flag possible mood disorders for referral purposes, and are likely to be an effective for targeting populations in need of mental health services that are in scarce supply in many countries (96) or specific health sectors (95). The inclusion of both psychiatric and somatic measures in interRAI assessments also allows clinicians to take into account the potential link between emotional problems and potential adverse physical health outcomes (97). By identifying clinically meaningful cut-points we can also flag opportunities for improving the quality of life and health with stepped approaches to management of depression (98).

A key opportunity with these mood scales is the value of having a common measure of mood disturbance that can serve to better integrate the identification and response to needs by different partner agencies and professionals in the continuum of care. These measures can be employed in multiple sectors and can follow them for longitudinal patient reported or clinician rated outcome monitoring as patients access different parts of the health system (40, 41). As has been the case with interRAI's existing care planning protocols, the threshold values identified for these scales can be used to trigger differential responses to indications of varying levels of severity or to change in the person over time (70, 82, 99–102). Hence, these scales lend themselves well to use in a measurement-based care (103) strategy whether using the self-report or composite version of the scale.

Several interRAI systems use the DRS or combinations of clinician rated mood items for risk-adjusted outcome-based quality indicators (32–34, 37–39, 73, 104). Although the present results are promising, additional research is needed to examine the responsiveness of the mood scale variants for use in performance measurement. From a face validity perspective, the ability to use the composite version of the scale should be appealing because it circumvents some of the limitations of clinician only ratings. Similarly, the use of the self-report scale for patient reported outcome measurement should be feasible in a very large range of settings where cognitive impairment is not severe. That raises the possibility of multi-sector outcome evaluations of the relative effectiveness of alternative approaches to the management of psychological wellbeing in at risk populations and in the general population.

There are several next steps that would be helpful in future research. Most obvious is the use of non-Canadian data to validate the psychometric properties of the scale variants in other countries, health systems, and populations. In addition, we did not have data for several newer interRAI instruments that have not yet been widely adopted in Canada, including the interRAI Intellectual Disability (105), interRAI Acute Care (106), interRAI Post-Acute Care (107). Moreover, we only examined adults aged 18+ in our study samples, so we are unable to comment on the performance of these scales in children and youth. interRAI has an extensive new suite of instruments for children and adolescents (5, 13, 14, 48, 108–110) so it will be important to establish the boundaries of where these scales do or do not function effectively.

Our present study has numerous strengths including large sample sizes (allowing for rich variation) in multiple sectors of the health system, population-level data for some settings, diversity of persons assessed in terms of clinical needs and demographic characteristics, and the use of trained health professionals to ensure good quality data. However, there are some important limitations to note as well. First, we have not yet examined the longitudinal, within-person trajectories of change in these scales to determine whether they are sensitive to both improvements and worsening of mood disturbance. Second, we need to consider their responsiveness to change when interventions are applied or their predictive validity for future events (e.g., new diagnosis, self-harm attempts, hospitalization). Third, it will be important to replicate these analyses with data from other countries and across cultural settings. Finally, for some settings we did not have access to validity indicators that were available elsewhere. These limitations can and should be readily addressed in future research. At this point we suggest that the level of evidence already available from this work supports adoption of these mood scale variants in jurisdictions that have already adopted interRAI systems.

## Data Availability Statement

The data from mandated use of interRAI systems may be obtained through a request of the Canadian Institute for Health Information. Access to data from the community surveys can be obtained on request from the lead author. Requests to access these datasets should be directed to hirdes@uwaterloo.ca.

## Ethics Statement

The studies involving human participants were reviewed and approved by University of Waterloo Office of Research Ethics. Written informed consent for participation was not required for this study in accordance with the national legislation and the institutional requirements.

## Author Contributions

JH drafted the first version of the manuscript and completed most analyses of data. JM and GB contributed to data analyses. JM, CP, JF, MS, GB, MF-M, HH, and SS made editorial changes to initial drafts. All authors contributed to the formulation of the ideas presented in the study and provided critical feedback to the manuscript. All authors contributed to the article and approved the submitted version.

## Funding

Portions of the community mental health, emergency department, and community survey datasets were gathered as part of research that received financial support from the Health Transition Fund - Health Canada, Canadian Frailty Network, and MITACS respectively.

## Conflict of Interest

The authors declare that the research was conducted in the absence of any commercial or financial relationships that could be construed as a potential conflict of interest.

## Publisher's Note

All claims expressed in this article are solely those of the authors and do not necessarily represent those of their affiliated organizations, or those of the publisher, the editors and the reviewers. Any product that may be evaluated in this article, or claim that may be made by its manufacturer, is not guaranteed or endorsed by the publisher.

## Footnotes

^1^Note: “home care” refers to community based personal support and nursing services provided in the person's home. “Community support service” agencies provide social and support services they tend to target a lower intensity population.

^2^The Resident Assessment Instrument 2.0 (RAI 2.0) is the predecessor to the interRAI LTCF. The RAI 2.0 is comprised only of clinician-rated items, so it cannot be used for the self-reported or composite variants of the mood scale. However, it does include the clinician-rated items that can be used for a clinician-only variant of the scale. Additional information is available on request.

^3^In this manuscript, “married” refers to both formalized legal marriages and common-law partners of either the same or opposite sex.

^4^Two options are used for anhedonia because some instruments use only one of those two items.
